# Intestinal Permeability and Drug Absorption: Predictive Experimental, Computational and In Vivo Approaches

**DOI:** 10.3390/pharmaceutics11080411

**Published:** 2019-08-13

**Authors:** David Dahlgren, Hans Lennernäs

**Affiliations:** Department of Pharmacy, Uppsala University, Box 580 SE-751 23 Uppsala, Sweden

**Keywords:** intestinal permeability, intestinal drug absorption, experimental and computational permeability methods

## Abstract

The main objective of this review is to discuss recent advancements in the overall investigation and in vivo prediction of drug absorption. The intestinal permeability of an orally administered drug (given the value P_eff_) has been widely used to determine the rate and extent of the drug’s intestinal absorption (F_abs_) in humans. Preclinical gastrointestinal (GI) absorption models are currently in demand for the pharmaceutical development of novel dosage forms and new drug products. However, there is a strong need to improve our understanding of the interplay between pharmaceutical, biopharmaceutical, biochemical, and physiological factors when predicting F_abs_ and bioavailability. Currently, our knowledge of GI secretion, GI motility, and regional intestinal permeability, in both healthy subjects and patients with GI diseases, is limited by the relative inaccessibility of some intestinal segments of the human GI tract. In particular, our understanding of the complex and highly dynamic physiology of the region from the mid-jejunum to the sigmoid colon could be significantly improved. One approach to the assessment of intestinal permeability is to use animal models that allow these intestinal regions to be investigated in detail and then to compare the results with those from simple human permeability models such as cell cultures. Investigation of intestinal drug permeation processes is a crucial biopharmaceutical step in the development of oral pharmaceutical products. The determination of the intestinal P_eff_ for a specific drug is dependent on the technique, model, and conditions applied, and is influenced by multiple interactions between the drug molecule and the biological membranes.

## 1. Introduction

Bioavailability is a key pharmacokinetic parameter that represents the fraction of an orally administered drug that reaches the systemic circulation in an uncharged molecular form (Equation (1)):

F = F_abs_ × (1 − E_G_) × (1 − E_H_) (1)

where F is the bioavailability, and E_G_ and E_H_ are the fractions extracted in the gut wall and liver, respectively. The fraction of the dose that is absorbed (F_abs_) and its absorption rate are largely determined by the following biopharmaceutical factors: the dissolution, solubility, luminal stability (chemical and/or enzymatic), intestinal transit time, and intestinal permeability of the active pharmaceutical ingredient (API). In order to achieve a sufficiently high systemic bioavailability, most drug products require pharmaceutical development to produce a plasma concentration-time profile that provides the optimal pharmacodynamic response and acceptable side effects. This is especially important for modified-release (MR) products, which are designed to improve the pharmacodynamic response. In general, oral products with poor bioavailability (F below 25%–35%) are recognized as having wider intra- and interindividual variability in plasma exposure (C.V. > 60%–120%) [1]. In 1996, Hellriegel et al. reported an inverse association between the bioavailability of oral drug products and the total variability of the bioavailability parameter. Now, more than two decades later, we know a little more about the reasons for poor and highly variable bioavailability values for oral pharmaceutical products. However, we still need to understand significantly more about the interactions between advanced oral dosage forms and the complex and dynamic gastrointestinal (GI) physiology of both healthy subjects and patients at all ages, from new-born to elderly, before these dynamic processes can be considered to be sufficiently understood [2]. It is crucial to obtain this knowledge so that it can be incorporated into sophisticated software that can then be applied in decision-making in drug development and regulatory work. 

To accomplish high bioavailability and low variability for oral pharmaceutical products, the API needs to be dissolvable and stable in the GI lumen, and also sufficiently absorbed at relevant sites in the small and large intestine. The regional intestinal effective permeability (P_eff_) is a key biopharmaceutical parameter that determines the absorption potential of the API from any dosage form [3]. Knowledge of the extent of drug absorption from the human large intestine is especially important for accurately predicting the manufacturing potential of a dosage form. The colon, as the final major organ in the GI tract, plays a key role in regulating diarrhea, constipation and the microflora composition, as well as delivery of drugs that are intended for prolonged release and administered once daily [4]. Although the regional intestinal P_eff_ is an important biopharmaceutical parameter, the final drug absorption profile for a drug in the intestinal tract is determined by the interplay of various processes such as motility, transit, solubility, dissolution, precipitation and stability. The Biopharmaceutics Classification System (BCS) of drugs provides information relevant to understanding and predicting GI drug absorption and bioavailability in general that is also relevant to the absorption potential for the colon [5,6].

**Figure 1:**
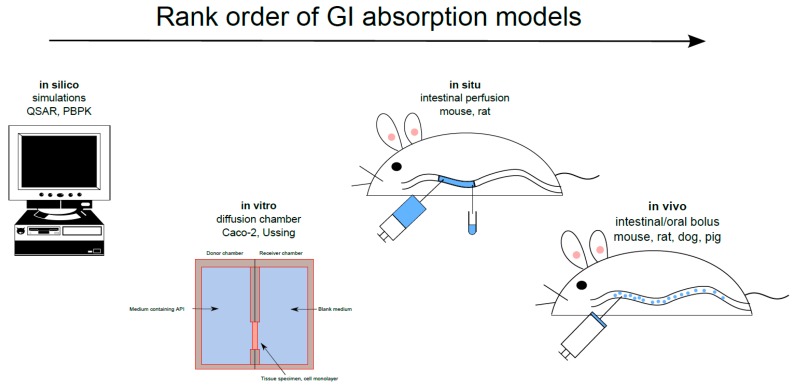
Gastrointestinal (GI) non-clinical absorption models ranked according to the order of their use in the drug discovery/development process for investigating transport mechanisms, determining intestinal permeability, and predicting plasma pharmacokinetic profiles. API = active pharmaceutical ingredient; PBPK = physiologically based pharmacokinetics; QSAR = quantitative structure-activity relationships.

There are many GI absorption models that investigate transport mechanisms, determine the P_eff_ and predict the plasma pharmacokinetic profile throughout the drug discovery/development process [7]. These models are often applied in the following order: in silico, in vitro, in situ and, most importantly, in vivo (Figure 1). In silico simulation of the absorption process from the GI tract has recently been used to optimize the API release rate, dose and dose distribution from the various release fractions in MR dosage forms. The accurate, reliable in silico prediction of GI absorption data for novel APIs and their dosage forms, vital for drug discovery and pharmaceutical product development, is a major challenge. Establishing an in vitro-in vivo link is also important, as emphasized in a recent report on patient-centric drug development from a product quality perspective [8]. Modeling and simulation approaches are used to characterize this in vitro-in vivo link with respect to the influence and clinical relevance of disease. Recently, eleven large pharmaceutical companies responded to a questionnaire regarding their use of in vitro and in silico biopharmaceutics tools for predicting in vivo outcomes. The companies are using these predictive models at various drug development stages, during regulatory contact for, for example, scientific advice, and for drug applications of various kinds [9]. Biorelevant dissolution-absorption physiologically based pharmacokinetic (PBPK) modeling and simulation were used by 88% of the responding companies in early drug development processes. The biopharmaceutical models were especially useful for investigating the impact of API particle size on intestinal drug absorption and for investigating different pharmaceutical dosage forms.

Extensive early human research has established that a good correlation exists between P_eff_ determined using the SPIP model and the F_abs_ from an immediate-release, dosage form [10]. Pharmacokinetic/mass-balance clinical studies are the best way of determining the fraction absorbed for an orally administered drug. However, these mass balance studies are very complex and expensive, as they require the API to be radiolabeled to enable validation of the drug and metabolite recovery [11]. Since the Food and Drug Administration (FDA) and European Medicines Agency bioequivalence guidelines use the F_abs_ to classify the permeation of drugs through the intestine in the BCS, the FDA BCS guidance committee have suggested using F_abs_ as a surrogate for P_eff_ [12].

The main objective of this review is to discuss recent advancements in the overall investigation and in vivo prediction of GI drug absorption. Intestinal P_eff_ has been widely used to determine the rate and extent of the intestinal absorption of orally administered drugs in humans. Among the various biopharmaceutical processes discussed, the focus of the review will be on intestinal permeability at different sites along the intestine. 

## 2. In Silico Gastrointestinal Absorption Predictions

In silico methods are now becoming widely used by the pharmaceutical industry and regulatory agencies to support decisions regarding dosage form development, bioequivalence and other bridging development processes. Pharmaceutical characteristics such as the particle size of the API and the coating layer, which affect the dissolution and subsequent intestinal absorption of the drug, and the plasma drug concentration-time profile are often applied [8]. Another common application for theoretical predictive software is to establish intestinal permeability and the quantitative structure-activity relationship (QSAR). These computer programs relate various molecular descriptors and physicochemical properties of the drug molecule (e.g., lipophilicity, the logarithmic acid dissociation constant pKa, hydrogen bonds, molecular mass) to crucial biopharmaceutical processes [13]. The success of a computational approach in predicting membrane permeability in the early high-throughput drug discovery phase is dependent on the statistical approach, the choice of molecular descriptors, and the quality of the experimental permeability data. The QSAR approach is consequently of limited use in the drug development process; it is primarily used for excluding molecules with obvious permeability limitations [14]. However, because of the increase in computer power, studies of drug permeation can now be performed using complex molecular simulations. These models can simulate the interaction between a molecule and a biological membrane, and thereby improve our mechanistic understanding of membrane transport [15,16]. Pharmaceutical scientists interested in developing MR dosage forms have focused their efforts on optimization of drug transport across biological membranes in the small and large intestines. For instance, the formation of intramolecular hydrogen bonds in the lipid bilayer, charge neutralization, and formation of zwitterions have been investigated for optimizing oral drug delivery through lipid bilayers. Alternatively, specific transporter proteins may be targeted by developing structural adaptations or by using a prodrug. In silica software programs for permeation models and hydrodynamic flow models based on chemical engineering approaches have been valuable in optimizing structure-activity relationships to retain key biopharmaceutical properties [17,18].

More complex in silico models are used to predict overall GI absorption and plasma drug concentration-time profiles following oral administration of drugs. These simulations depend on API-specific physiochemical properties, such as solubility and logarithmic distribution coefficient (log D), and other drug parameters, such as disintegration and dissolution rates, physiological parameters (e.g., intestinal pH, transit times, and morphology), flow characteristics, and the drug first-pass effects in gut and liver, as well as subsequent disposition in vivo [19]. Computer simulations should ideally integrate experimental in vitro and in vivo data to increase their accuracy [20]. However, the accuracy of these models in predicting the fraction absorbed from well characterized physicochemical and biopharmaceutical factors is currently too low to compete with experimental in vitro and in vivo studies in drug development [21]. Nonetheless, a validated in silico model could be useful for evaluating, for instance, the impact of changes in drug formulation or drug-drug and food-drug interactions, which could help guide the design of both preclinical studies (for instance, toxicokinetic studies for safety evaluation) and clinical studies [22].

The full results of the survey by Flanagan et al. in 2016 revealed that biorelevant dissolution testing in simulated media and physiologically based dissolution and PBPK studies are widely used for oral drug product development by the European Federation of Pharmaceutical Industries and Associations (EFPIA) participants in the Innovative Tools for Oral Biopharmaceutics (OrBiTo) project, to investigate the interplay between various biopharmaceutics factors [23]. When in vitro dissolution investigation is introduced in the projects, 80% of the companies use biorelevant dissolution media (SGF, FaSSIF, FeSSIF) in the first step, prior to using simplified buffers for BCS class II and IV APIs. In addition, the survey indicated that these data are seldom presented to regulators. Approximately 70% of companies seldom or never submit these biorelevant dissolution data at the investigational new drug stage, and the corresponding fraction at the new drug application/marketing authorization application stage is 60%. The potential usefulness of in vitro dissolution studies performed in biorelevant media for quality control release testing was also considered. Three PBPK software packages (GI-Sim, Simcyp^®^ Simulator, and GastroPlus™) were tested and compared within the OrBiTo project during a blinded “bottom-up” study of human pharmacokinetics. It was found that the bioavailability of orally administered APIs that permeated the intestine poorly was underpredicted, probably because accurate and physiologically relevant estimates of the intestinal surface area, the absorption properties from the large intestine, and/or the role and importance of transport-mediated intestinal permeation were not available [24,25,26]. The bioavailability of APIs with acidic pKa was underpredicted, possibly because of underestimation of intestinal permeation (role of ionization and transport-mediated absorption) and/or underestimation of the luminal solubilization of weak acids as a result of less-than-optimal intestinal pH settings or underestimation of the bile micelle contribution. The bioavailability of weak bases was overpredicted, suggesting inadequate models of luminal precipitation or absence of in vitro precipitation information. The relative bioavailability of both highly hydrophobic compounds and poorly aqueous-soluble APIs was underpredicted, suggesting inadequate models of solubility/dissolution, underperforming bile dissolution enhancement models and/or lack of biorelevant solubility measurements. These results clearly identify areas for improvement in theoretically based software, modeling strategies, and production of relevant experimental input data. 

One emerging area in the in silico prediction of fraction absorbed and bioavailability that has gained regulatory interest and is being prioritized to justify product specifications or formulation/process changes is the use of integrated in silico PBPK absorption models in combination with high quality biopharmaceutical in vitro data [19,27]. For instance, the in silico approach may be useful for demonstrating the bioequivalence of different formulation concepts, defining the API and formulation design space and manufacturing controls, anticipating post-approval manufacturing changes and obtaining biowaivers.

## 3. Gastrointestinal Experimental Absorption Models

Preclinical GI absorption models are currently in demand for the pharmaceutical development of novel dosage forms and new drug products. However, we need to improve our understanding of the interplay between pharmaceutical, biopharmaceutical, biochemical and physiological factors in determining the fraction absorbed and bioavailability before reliable models can be developed. Currently, our knowledge of GI secretion, GI motility and regional intestinal permeability, in both healthy subjects and patients with GI disease, is limited by the relative inaccessibility of some intestinal segments of the human GI tract [28]. Conventional clinical approaches of exploring and collecting GI content remain invasive, resource intensive, and often unable to capture all the information contained in these heterogeneous GI samples. A new class of GI sampling capsules is available, which is based on an intra-luminal technique that offers the possibilities of the spatial and temporal information of the GI samples [29]. The future use of these clinical techniques in oral biopharmaceutics expects to improve our understanding of the GI processes involved in oral drug delivery. Our understanding of the complex and highly dynamic physiology of the region from mid-jejunum to the sigmoid colon in particular could be significantly improved. One approach to the assessment of intestinal permeability is to use animal models that allow these intestinal regions to be investigated in detail and then to compare the results with those from simple human permeability models such as cell cultures.

### 3.1. In Situ

The various in situ models for determining P_eff_ are often based on disappearance of the drug from a defined perfused intestinal segment. The selected intestinal segment may be continuously perfused, as in the single-pass intestinal perfusion (SPIP) model, or be closed off, as in the closed-loop Doluisio model [30]. Intestinal P_eff_ is calculated in different ways depending on the hydrodynamics in the specific model. 

The SPIP model is generally used after the drug discovery phase and in the early formulation development stage of drug development, when more relevant biopharmaceutical data are needed. One major advantage of the SPIP model is that it enables relevant mechanistic investigations of drug absorption and anticipates the effects of various physiological processes. Some of the advantages of the SPIP model over in vitro models are the intact intestinal morphology, the presence of blood flow, the presence of neural and hormonal feed-back mechanisms and the possibility to control luminal conditions [31].

**Figure 2:**
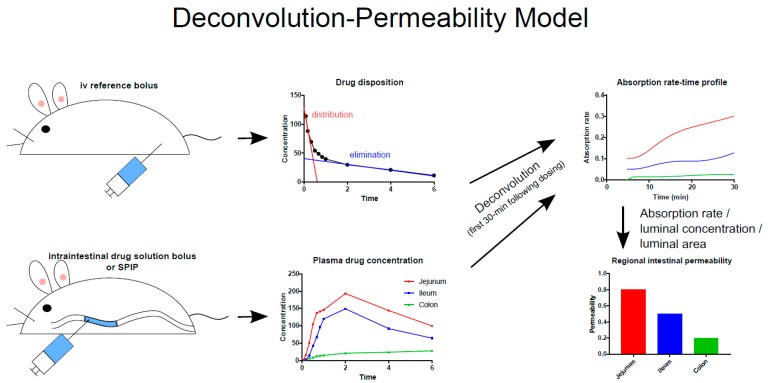
Schematic illustration of the deconvolution-permeability model, which can be used to determine the regional intestinal permeability of model drugs based on their appearance in the plasma following intravenous and intraintestinal administration of the drug in solution [36]. The method has been successfully applied to determination of intestinal permeability in rats, dogs, and humans [3,31,33,35,37,38]. SPIP = single-pass intestinal perfusion.

The rat SPIP model is commonly used to investigate GI physiology, membrane drug transport, and the potential for a new drug candidate to be formulated in an oral MR dosage form. The potentially negative effects of abdominal surgery in this model are reduced by concomitant treatment of the rats with parecoxib, a selective cyclo-oxygenase-2 inhibitor that has been shown to positively affect some intestinal functions such as GI motility, epithelial permeability, fluid flux, and ion transport [32,33,34]. However, in a recent SPIP study, treatment with parecoxib had only minimal effects on membrane permeability and water flux [35]. It was also established that the permeability of the intestine to poorly permeating drugs is best determined on the basis of the appearance of the parent drug in plasma rather than the disappearance of the drug from the perfused intestinal segment (Figure 2) [35]. A study by Dahlgren et al. in 2019 also clearly showed that when the intestinal P_eff_ is estimated using luminal disappearance, it should include negative values in the calculation to increase the accuracy of the final P_eff_ [35].

### 3.2. In Vivo

Classical in vivo single-dose pharmacokinetic models in which drug solutions or formulations are administered orally, or directly into the stomach or intestine in suitable animal species, may also be used to investigate the P_eff_, the fraction absorbed and the bioavailability. In such studies, the value for the fraction absorbed includes the impact of other biopharmaceutical processes such as dissolution, precipitation, transit, etc. [37]. These in vivo animal models are the most clinically relevant because physiological factors, such as gastric emptying time, luminal water content and drug degradation, and post-absorption first-pass metabolism affect the determined parameters and the predicted outcome. These types of models are obviously less applicable for mechanistic studies of intestinal absorption, as the relative impact of the different factors can be difficult to assess in detail. 

When using these in vivo GI models, motility is defined as movements of the GI tract that cause mixing and transit of luminal chyme above the absorptive and secretary intestinal surface. These mixing and transit processes are located both in the lumen and in the area adjacent to the intestinal epithelium, and are coordinated and regulated through a complex circuitous interaction between a number of physiological systems including, but not limited to, the enteric, autonomic, and central nervous systems. It has been suggested that long-distance and short-distance motor activities in the GI tract could interact to propel undigested luminal chyme along the tract, where regional mixing promotes intestinal absorption [39]. If disturbances occur in any of these systems, it could disrupt the coordination of the propulsive peristalsis, potentially leading to dysmotility and ultimately various GI-specific symptoms. The relevance of these motility patterns to the intestinal absorption of drugs and nutrients is an important research topic for the future.

It is also crucial to consider the effects that these GI digestive processes may have on the intestinal absorption of drugs from different formulations and the local effects of some drugs with targets in the lumen (luminal enzymes such as lipases and α-amylases) or receptors on the luminal side of the epithelium. When isolated from the central nervous system, the gut is the only organ that has integrative neuronal activity. This activity may be stimulated by luminal contents that act as specific sensory transducers on certain specific epithelial cells, such as enterochromaffin cells, which release 5-hydroxytryptamine. 5-hydroxytryptamine stimulate intrinsic and extrinsic primary afferent neurons that are present in both the submucosal and myenteric plexuses. The role of integrative neuronal and local endocrine effects on intestinal absorption needs to be better understood.

### 3.3. In Vitro

Common in vitro models for studying membrane permeability include monolayers of cells grown on cell culture filters (e.g., Caco-2 cells), and excised intestinal tissue samples mounted in a diffusion (Ussing) chamber. The apparent permeability (P_app_) is calculated by relating the mass of the drug appearing in the receiver chamber at multiple time points (dM/dt) to the area of the barrier (A), and the drug concentration in the donor chamber (C_donor_) [40,41,42]. The intestinal P_app_ is an intrinsic constant associated with a molecule that relates the flux to the concentration gradient; it can therefore be used to predict drug transport over any type of biological cell barrier by adjusting for, for instance, area, hydrodynamics and the pH of the medium. In addition, the controlled aqueous conditions in a cell-based in vitro system offer the possibility of performing mechanistic transport investigations if the expression and function of the involved proteins are accurate [43,44]. The Ussing chamber system enables regional intestinal permeability [7]. Limitations associated with these models include the high inter- and intra-laboratory variability, and sensitivity of the cell/tissue to the preparation setup and chamber media. For permeability investigations in drug discovery, it is therefore recommended that relative P_app_ values (compared to reference standards) be used, instead of absolute P_app_ values [45]. The BCS can also be used to predict in vivo drug absorption based on in vitro drug dissolution data [6]. It is also well established that these systems are more sensitive for pharmaceutical excipients and enhancers with intended absorption-modifying properties. 

One recent and exciting advancement of an in vitro intestinal absorption models is intestinal organoids [46]. Organoid technology from various species bridges the gap between conventional two-dimensional cell line culture and in vivo models [47,48,49]. One of the objective with this in vitro approach is to improve organ development and accordingly improve the in vivo relevance. Intestinal organoids is expected to become a useful drug development technology for various biopharmaceutical and pharmacokinetic analysis and in vivo predictions. 

## 4. Intestinal Membrane Transport

The movement of ions, transmitter compounds, nutrients and other endogenous substances across various biological membranes is a central dynamic molecular process that is essential for life in mammals. Selective permeability is a key feature of biological membranes and is determined by the physicochemical properties of the lipid bilayer and the channel-forming membrane proteins together with the physicochemical properties and molecular structure of the drug molecules. These transport processes across biological membranes with a diverse composition occur via direct and indirect energy-demanding carrier-mediated (CM) mechanisms even against a concentration gradient. Facilitated membrane diffusion, passive membrane diffusion and paracellular diffusion occur along a concentration gradient. Biological membranes encapsulate cells and their contents to optimize the various functions that cells are responsible for in a living organism. At the core of any biological membrane is a lipid bilayer, which in vivo can be composed of hundreds of different types of lipid molecules. Membrane lipids have amphiphilic molecular properties with a polar head group and a non-polar tail comprising esterified fatty acids. These lipid molecules vary widely in terms of size, chemical structure and polarity and can be combined and assembled to provide a wide variety of physical properties and functions.

**Figure 3:**
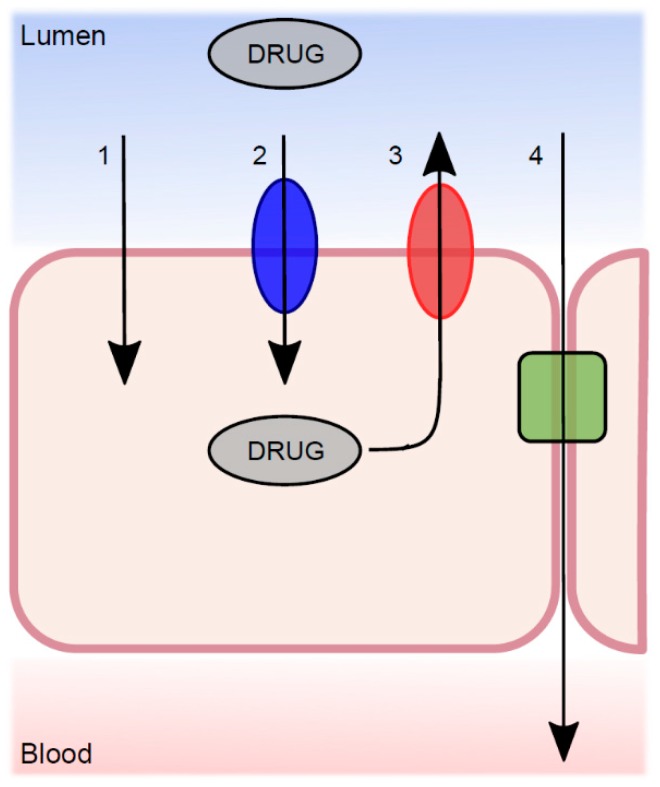
The transport mechanisms from the lumen across the intestinal epithelium, which determine the net permeability of a luminally dissolved drug molecule. (**1**) Passive transcellular diffusion; (**2**) absorptive carrier-mediated transport; (**3**) efflux carrier-mediated transport; and (**4**) passive paracellular diffusion.

Movement of drugs across various membranes is essential for many pharmacokinetic and pharmacodynamic processes. The basic nature of drug transport is divided into transcellular and paracellular processes, where the transcellular route is the most common (Figure 3). Transcellular transport, either passive diffusion or CM, occurs across the intestinal cell (enterocyte), through both the apical and basolateral membranes. Paracellular transport occurs between the epithelial cells. During the last decade, a large number of published articles have discussed the existence and role of passive diffusion across biological membranes as a relevant mechanism [50,51,52,53]. The overall conclusion is that passive transcellular diffusion is the predominant mechanism for transfer of drug substances, but that this co-exists with CM trans-membrane processes.

The pH partitioning theory states that the charged species of a weak acid or base do not contribute to passive lipoidal diffusion across the cell lipid bilayer, as they do not partition into octanol [54]. The permeation of these molecules is highly dependent on the pH at the surface of the lipid cell membrane and the pKa of the drug [20]. This has been experimentally illustrated in the Caco-2 cell monolayer model, where the transport of alfentanil and cimetidine was linearly correlated to the un-ionized fraction (i.e., the pH) [55]. The pH also affects the transport of propranolol in Caco-2 cells, MDCK cells, and the rat Ussing chamber; reducing the pH from 7.4 to 6.5 in the donor compartment reduces the transport of this low molecular mass (259.3) basic drug [56]. 

**Figure 4:**
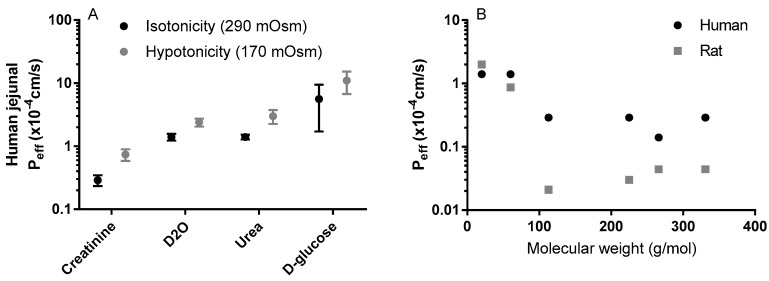
(**A**) The influence of luminal tonicity on the effective permeability (P_eff_) of human jejunum to four model compounds with different molecular masses: D_2_O 20 g/mol, urea 60 g/mol, creatinine 113 g/mol, and D-glucose 180 g/mol [58]; (**B**) The influence of the molecular mass of six passively absorbed compounds on the human and rat jejunal P_eff_ values: D_2_O 20 g/mol, urea 60 g/mol, creatinine 113 g/mol, terbutaline 225 g/mol, atenolol 266 g/mol, furosemide 331 g/mol [58]. Figures are remade based on historical data.

The concept of the pH partitioning theory for predicting passive membrane transport of drugs and other xenobiotics is, however, not that straightforward [54]. This is illustrated by the permeation of a charged species across cell barriers in the water-filled paracellular pores, a process which is typically faster for smaller (molecular mass less than approximately 250) and longer molecules [57,58]. These paracellular pores can also have different charge-selectivity, based on the claudin proteins (a large family of proteins that modulate paracellular permeability [59,60]. More research is required on the mechanisms that underlie differences in paracellular absorption for drugs of different sizes (g/mol), both within and between species (Figure 4A,B) [58,61]. Although we have some information on the roles of individual claudins, some of which are thought to form charge- and size-selective tight-junction pores for smaller molecules, relatively little is known about their interactions [62]. Further, the permeation of charged anions through the lipoidal membrane can be many times more rapid than expected, controlling membrane transport at all in vivo-relevant pHs [54]. This must be taken into consideration to avoid overestimation of the fraction of a compound that is transported across the paracellular route [63]. Two extensively permeating compounds, ketoprofen and metoprolol, are, for example, rapidly absorbed across human and rat intestinal mucosal barriers, where the pH is between 6.5 and 7.4 and only about 0.1 to 1% is in the neutral form [3,64]. This is despite the pH-dependent decrease in ketoprofen permeation observed when increasing pHs in a parallel artificial membrane permeability assay [65]. In addition, quaternary ammonium compounds also permeate lipoidal membranes to different degrees, despite their permanent charge [65,66].

Hence, it is obvious that the pH partitioning theory alone cannot be used to predict the passive lipoidal diffusion of compounds. Several non-CM transcellular transport mechanisms have consequently been proposed to account for the transport of charged and/or hydrophilic drug molecules (as well as other xenobiotics) across the lipoidal membrane. Two mechanisms, based on molecular simulations and membrane experiments, propose the creation of water pores, or lipid head-group pores [67]. Water pores are thought to exist because water has been shown to be present in the assumed water-free membrane core [68]. This water reduces the energy cost of a hydrophilic drug dissolving in the lipoidal membrane, as the need for molecular dehydration is reduced. The total cost for a drug dissolving in the lipid membrane is hence lower than would be expected. Lipid head-group pores are assumed to be formed by an interaction of ions or the drug doxorubicin with the lipid head groups [69,70]. These head-group pores would then facilitate the transport of charged and hydrophilic compounds. 

An additional theory is that transmembrane transporter proteins increase the transport of small hydrophilic molecules by facilitating transport along the exterior [71]. This would not, however, explain the substantial transport of charged molecules over protein-free lipoidal membranes. The transport of charged molecules by co-permeation with a counter ion is also a possibility [72,73]. However, given the rapid transport of, for instance, ketoprofen in vivo, and the limited effect of ion pairing with non-organic ions, ion pairing seems a less likely mechanism behind the substantial absorption of some charged drugs in vivo [3,73,74]. 

Among the molecular descriptors evaluated by Lipinski (e.g., polar surface area, hydrogen bond donors (HBDs)/acceptors, Log D), the number of HBDs is the most restrictive when it comes to intestinal membrane transport/absorption [18,75]. Two drugs breaking this rule (i.e., >5 HBDs and high fraction absorbed), tetracycline and rifampicin, were recently analyzed to evaluate their potential for crossing the intestinal membrane by passive lipoidal diffusion, regardless of their unfavorable properties [67]. A liposomal permeation assay showed that rifampicin and metoprolol permeated to a similar extent, and that tetracycline and labetalol permeated similarly, suggesting that these >5 HBD drugs can be absorbed by passive lipoidal diffusion to a substantial degree. 

To explain why some drugs are absorbed by passive lipoidal diffusion, regardless of their unfavorable physicochemical properties, it is necessary to find more complex descriptions of the molecular interaction with the lipoidal membrane. Permanently charged molecules, for instance, vary in their degree of passive permeation according to their ability to spread the charge over several ring structures [66]. Several experimental studies (based on nuclear magnetic resonance and the crystalline form) have also shown that intramolecular hydrogen bonding can mask polar structures and thus increase membrane transport [76,77]. The principle is that the intramolecular hydrogen bonding reduces the thermodynamic penalty of dissolving in the membrane core [15]. 

This has also been shown in several molecular dynamics simulations of the transport of solutes across a lipid bilayer. A drug usually loses degrees of freedom when dissolving in the membrane core. The energy demand is reduced by intramolecular hydrogen bonding and with lipid head groups. By changing the type of intramolecular hydrogen bonding in β-blockers, the molecular conformation can be changed, depending on its position in the membrane bilayer. For instance, a more elongated shape is favored in the center of the lipid bilayer and a more folded structure is favored at the interface. The more elongated, flexible shape allowed in the center favors a flip flop to the other side, while also generally reducing the cost of dehydration, when dissolving in the bilayer by forming intramolecular hydrogen bonds [67,68]. Tetracycline is thus able to hide three of the six hydrogen donor groups by intramolecular hydrogen bonding, as shown experimentally by high-intensity synchrotron radiation [78].

The accuracy of QSAR predictions of intestinal absorption, based solely on the physicochemical descriptors of a molecule, is also significantly improved by including molecular dynamics simulations [79]. Molecular simulations have also been successfully used to predict the effects of cholesterol in the lipid membrane; cholesterol typically makes the bilayer more stiff and less permeable (also described as reduced membrane fluidity) [15]. Molecular simulation investigations have also been able to replicate experimental data on the relative permeation of a set of compounds (atenolol < pindolol < progesterone < testosterone), based on free energy transfer in different depths of the membrane bilayer [80]. 

The detailed discussion of the intestinal membrane transport of atenolol below is based on data from various sources, ranging from theoretical calculations to human pharmacokinetic data.

## 5. Atenolol

Transport mechanisms for a low molecular mass drug is often interpreted based on multiple techniques. Atenolol is a well-recognized BCS class III drug that has been proposed to be transported by transcellular, paracellular as well as with various CM processes (see below). Atenolol is therefore suitable for illustrating the complexity of classifying a drug’s transport mechanisms, as data from various in silico, in vitro, in situ, and in vivo models are needed. 

**Figure 5:**
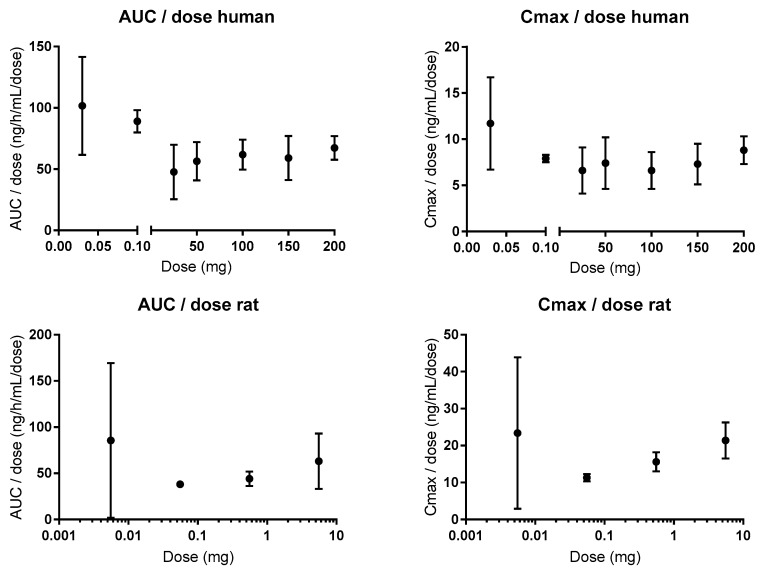
Dose proportionality in the area under the concentration-time curve (**AUC**) and maximum concentrations; (**Cmax**) of atenolol in humans (0.1–200 mg) and rats (0.55 µg–5.5 mg) [81,82,83,102]. Figures are made based on historical data.

Following oral administration to humans, the plasma pharmacokinetics of atenolol are linear for doses of 25 to 200 mg for the area under the concentration-time curve (AUC), and for oral doses of 0.1 to 200 mg (1.4–2857 µg/kg) for the maximum concentration (Cmax) (Figure 5) [81,82,83]. There is a 1.5- and 1.6-fold higher AUC for doses of 0.03 and 0.1, respectively, and a 1.6-fold higher Cmax for the 0.03 mg oral dose, than for the average values in the clinical oral dose range [81,84]. These data from microdosing studies (0.03 and 0.1 mg) mean that there is some CM contribution to the intestinal permeation of atenolol at lower oral doses/luminal concentrations. Xenopus laevis oocyte transport studies suggest that OATP1A2 might be a plausible absorptive transporter for atenolol [85]. However, it should be mentioned that there was no statistical difference in AUC between 0.1 and 50 mg in one of the microdosing studies, which suggests that passive and non-saturable transmembrane transport might prevail in vivo for atenolol [81]. In addition, the difference in plasma exposure is unrelated to the elimination of atenolol, which is 100% renal (of parent drug) in both humans and rats, and is unaffected by oral doses in the range of 0.3–80 mg/kg [86,87,88]. Consequently, the dose of atenolol does not affect its renal clearance, which has been shown to be partly mediated by the efflux transporters OCT2 and MATE 1 and 2 [89]. This is also in accordance with their Km values (280, 32, and 76 µM, respectively), which are substantially higher than the maximum plasma concentration of 2 µM following an oral dose of 100 mg [88].

In humans, co-administration of oral atenolol with apple or orange juice in the fasted state decreases the plasma exposure of atenolol to 20–50% of that observed with water [90,91]. This interaction may be the result of inhibition of absorptive transporters, as observed for fexofenadine and celiprolol [92]. However, given the large volumes of apple juice (600–1200 mL) or orange juice (200 mL) used, and the notoriously high osmolarity of fruit juices, the reduced exposure is probably the result of an increased intestinal transit time. Similar results have been observed for oral atenolol when administered with a non-absorbable osmotic load (500–700 mOsm); the intestinal transit time was decreased from 180 to 60 min, and exposure was decreased from 1.7 to about 0.4 mg × h/L, compared to water [93]. 

Efflux ratios (B–A:A–B) of 2.3 and 3.5 were observed for atenolol in cell monolayer studies (Caco-2 and IPEC-J2); these were reduced to 1.7 and 1.1 with coadministration of the Pgp inhibitors verapamil and zosuquidar, respectively [94,95]. This suggests that atenolol might be a Pgp substrate. However, other Caco-2 studies (atenolol concentrations between 30 µM and 3.8 mM) have shown that the efflux ratio of atenolol is 1, is concentration independent, and differs between laboratories (ranging from 0.18 to 3.76) and between batches in the same laboratory [44,96,97]. The P_app_ of atenolol was also unaffected by verapamil in the mouse SPIP model, and after knockout of the Pgp gene [98,99]. Similarly, the absorption rate of atenolol was increased in the rat in an in situ jejunal loop study with co-administration of another Pgp inhibitor, cyclosporine [100]. In addition, atenolol has linear pharmacokinetics (AUC, Cmax) in rats following oral administration of doses between 0.55 µg and 5.5 mg (0.167–1670 µg/kg), and oral co-administration of the Pgp inhibitor itraconazole to humans did not affect its pharmacokinetics (Figure 5) [101,102].

**Figure 6:**
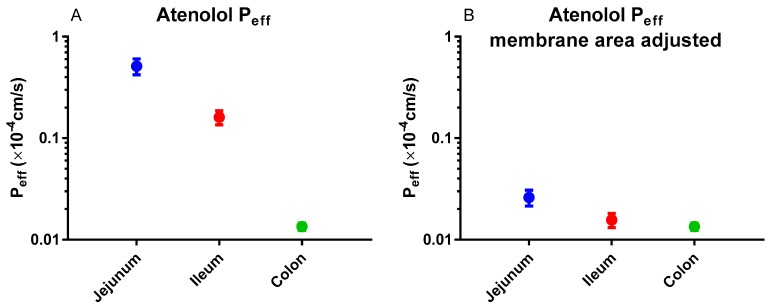
(**A**) Regional intestinal effective permeability (P_eff_) of atenolol in humans [3]. (**B**) Surface area (villi and folds)-adjusted regional intestinal P_eff_ values for atenolol in humans: jejunum 19-fold, ileum 10-fold, colon 1-fold [103]. Figures are remade based on historical data.

In humans, the regional intestinal P_eff_ for atenolol was substantial (Figure 6A) [3]. However, this difference almost disappeared when the P_eff_ value was corrected for the regional intestinal difference in surface area (Figure 6B) [103]. These results indicate that passive membrane permeation is the predominant transport mechanism of atenolol.

Atenolol has generally been regarded as a passive permeability marker in blood-brain barrier (BBB) transport studies, based on the linear plasma clearance of the drug into the brain over time [104,105]. However, its use as a marker for passive permeability in the brain and intestines has been questioned recently [106]. based on the free fraction of atenolol in the brain extracellular fluid (3.5% of that in blood plasma at steady state), suggesting CM efflux of atenolol from the BBB by an unknown transporter protein. The paper did not address why the data from a rat study evaluating BBB transport should be valid for the intestine, however.

In summary, there are conflicting data regarding the contribution of CM transporters to the intestinal absorption of atenolol. Cell-based assays have indicated an affinity for efflux proteins, but one of two oral microdosing studies indicated an affinity for influx proteins. However, taking into account all the available data, including extensive oral plasma pharmacokinetic data from a wide dose range (0.1–100 mg), it seems likely that the influence of intestinal transporters on the intestinal absorption of atenolol is, at most, modest. Atenolol can be considered to be transported almost exclusively by the passive route (lipoidal and/or paracellular), especially at oral human doses > 1 mg (>14 µg/kg), representing an intestinal concentration of about 20 µM (1 mg in 250 mL).

## 6. Conclusions

Investigation of intestinal drug permeation processes is crucial for the development of oral pharmaceutical products. The prevailing hypothesis for the permeation of drugs through the intestine involves several parallel CM and passive permeation mechanisms (such as passive lipoidal diffusion, CM uptake transport, CM efflux, paracellular diffusion, mucus resistance, endocytosis and transcytosis). The determination of an intestinal P*_eff_* for a drug is based on the technique, model and conditions applied and is influenced by the multiple interactions between the drug molecule and the biological membrane. Further development of the oral biopharmaceutics system requires the development of novel in vitro models and the use of human and animal in vivo techniques. For instance intestinal organoid technologies that bridge the gap between conventional two-dimensional cell line culture and in vivo models are expected to improve our mechanistic understanding. These innovative and more complex in vitro models need extensive comparison to high-quality in vivo data. Novel clinical techniques are expected to provide an improved understanding and high-quality data of biopharmaceutical relevant GI processes.

## Abbreviations

API	Active pharmaceutical ingredient
AUC	Area under the concentration-time curve
BBB	Blood-brain barrier
BCS	Biopharmaceutics classification system
CM	Carrier-mediated
C_max_	Maximum concentration
F	Bioavailability
F_abs_	Fraction absorbed
FDA	US Food and Drug Administration
GI	Gastrointestinal
HBD	Hydrogen bond donor
MR	Modified-release
OrBiTo	Innovative Tools for Oral Biopharmaceutics
P_eff_	Effective permeability
PBPK	Physiologically based pharmacokinetic
QSAR	Quantitative structure-activity relationship
SPIP	Single-pass intestinal perfusion
